# Crosstalk Between Protein Restriction and Fasting and Its Impacts on Growth, Digestive Enzymes, Immunity, Antioxidant Activity, and Relative Genes of Whiteleg Shrimp (*Litopenaeus vannamei*)

**DOI:** 10.1155/anu/6398266

**Published:** 2025-02-11

**Authors:** Fatemeh Jahangiri, Ebrahim Sotoudeh, Ahmad Ghasemi, Noah Esmaeili

**Affiliations:** ^1^Department of Fisheries, Faculty of Nano and Bio Science and Technology, Persian Gulf University 75169, Bushehr, Iran; ^2^Persian Gulf Research Center, Persian Gulf University 75169, Bushehr, Iran; ^3^Guangdong Provincial Key Laboratory of Marine Biotechnology, Shantou University, Shantou, China

**Keywords:** compensatory growth, feed conversion ratio, feeding strategies, growth performance

## Abstract

Feed strategies such as compensatory growth and protein restriction have been applied to optimize growth and feed efficiency in aquatic species. The effects of protein restriction (from 1 to 4 weeks of feeding with dietary 35% protein) and 1 week of fasting on growth, body composition, hemolymph parameters, digestive enzymes, serological enzymes, immune and antioxidant system, and relative gene expressions in whiteleg shrimp (*Litopenaeus vannamei*) (0.30 ± 0.03 g) were investigated. Treatments were experienced in 1 out of 8 weeks of fasting but not in the Control and 35%P groups. Other groups were 7P40 (1-week fasting), 6P40 (1-week feeding dietary 35% protein), 5P40 (2-week feeding with dietary 35% protein), 4P40 (3-week feeding dietary 35% protein), and 3P40 (4-week feeding dietary 35% protein). The results indicated that there was no difference in weight gain among Control (10.22 g), 7P40 (9.37 g), and 6P40 (9.27 g) groups. Feed efficiency in 35%P was significantly lower than in Control. The 5P40, 4P40, 3P40, and 35%P treatments had lower protein and lipid contents in the body, protease, total protein, and cholesterol compared with the Control. Immunity and antioxidant systems were suppressed by the application of fasting and protein restriction simultaneously so that acid phosphatase, lysozyme, superoxide dismutase, glutathione peroxidase, and lysozyme gene in 4P40, 3P40, and 35%P treatments were lower, and also, these groups had higher alanine aminotransferase levels than the Control. In conclusion, this study suggests that applying both protein restriction and fasting impairs the growth and health of whiteleg shrimp, and at least 6 out of 8 weeks, whiteleg shrimp should be fed with a dietary 40% protein.

## 1. Introduction

Whiteleg shrimp (*Litopenaeus vannamei*) is the most farmed shrimp species, with an annual production of 5.8 million tonnes in 2020 [1]. This species' farming is in the way of sustainability as mariculture and coastal aquaculture have been the best options to fulfill food demands in the upcoming decades [2]. According to Fao [1], over 55% of total aquaculture production is derived from saline water. One of the main concerns of aquaculture is providing high quality and quantity of protein for farmed aquatic species. For example, whiteleg shrimp required at least 35% protein as the optimum level [3]. In whiteleg shrimp aquaculture, 40%–60% of production cost comes from feed cost, most of which comes from protein. There have been several approaches to using less protein, especially expensive ingredients, such as alternative ingredients and nutrient levels or feeding strategies. Feeding strategies play a key role in improving growth performance and feed efficiency and reducing waste and feed and labor costs. However, compared to, for example, fish meal replacement studies, less attention has been paid to this approach. Various feeding strategies can be effective depending on species, size, and farm conditions, such as ration change, one-off days feeding, feeding frequency, feed restriction, changing nutrient components, and protein restriction.

Feeding restriction, fasting, or feed deprivation are commonly used in those systems with environmental issues and stressful situations in which animals are not able to get feed at optimum levels. Aquatic species experience feed restrictions in their natural environment when feed availability varies from time to time, and evolutionary, their body has been adapted physiologically to tolerate these conditions. Shrimp can mobilize body reserves during fasting to provide energy for metabolism. This energy can be provided by lipid breakdown in the liver and adipose tissue, muscle protein, and the breakdown of hepatic and/or muscle glycogen into glucose as an energy substrate [4]. When feed is available again, animals increase food consumption to quickly recover their body reserves, which is called compensatory growth. Different physiological changes occur accordingly, such as hematology and blood biochemistry, immune system and antioxidants, osmoregulation, gene expression, and almost all physiological parameters depending on species, size, and fasting period. In shrimps, several studies investigated feed restriction and compensatory growth. Some of them found up to 25% reduced ratio in shrimps compared to satiation level did not decrease growth and survival rate and improved feed efficiency and digestive enzyme activities [5–10]. Short fasting for up to 7 days did not negatively impact the growth, feed efficiency, and health of shrimps and was used as a trigger for total compensatory growth [11–17]. Also, studies on protein restriction in shrimp showed feeding animals with 30% protein for 2 weeks and then feeding with 45% protein for 4 weeks did not impair growth, feed intake, and feed efficiency [18]. Further, various feeding weekly schedules with dietary 43% and 36% protein (restricted diets) did not decline weight gain, feed efficiency, digestive enzyme activities, and crude protein content [19]. Even though these studies were in shrimps still, the output varied due to the severity and duration of protein and feed restriction, experimental conditions, size, species, diet, and interspecific differences.

Previous experiments in animals have not tested how protein restriction during the realimentation phase can affect final growth rate and health. The question is whether animals respond differently to dietary protein levels during compensatory growth. In other words, how using fasting and protein restriction simultaneously can affect the growth, feed efficiency, and health of animals. Whiteleg shrimp, as the most produced crustaceans, was selected as the model for this study. We hypothesized that the protein restriction during the compensatory phase could negatively impact growth, feed efficiency, flesh quality, digestive enzymes, immune system, antioxidant activity, and gene expression. Therefore, the objective of this research was to investigate how protein restriction and compensatory growth can together affect growth performance, feed efficiency, survival rate, body composition, hemolymph parameters, immune system, antioxidant activity, and relative gene expressions of whiteleg shrimp.

## 2. Material and Methods

### 2.1. Ethics Statement

The stress experienced by whiteleg shrimp during handling was minimized by following the guidelines established in the Declaration of Helsinki (1975), the Society for Animal Care and Use Guidelines (1998), and the national ethical framework for animal research in Iran [20].

### 2.2. Shrimp, Husbandry Trial, and Experimental Design

This research was conducted at the Laboratory of Aquatic Research, Persian Gulf University, Bushehr, Iran. Shrimp were obtained from a local farm company and fed a starter diet (The Behsan Taghzieh Arian Company [BTA Group] [pellet size: 0.8–1.4 mm]) for a period of 14 days. Prior to the experiment, shrimp were visually examined for any evidence of disease. Only healthy individuals were selected and then transferred to experimental tanks [21]. A total of 525 whiteleg shrimp (0.3 ± 0.03 g) were stocked in 21 tanks (300 L, diameter: 90 cm with 200 L water volume) with 25 shrimp per tank in triplicate. The tanks received filtered and disinfected seawater treated with chlorine (salinity: 40 ± 0.6 ppt), with ~20% of the water being replaced each day [21]. The water quality parameters were measured, so the average temperature and pH (Hanna, HI 98128, USA) were 29.0 ± 1.1°C and 7.5 ± 0.5, respectively; and also, the total ammonia nitrogen (Desun uniwill, China) was 0.08 ppm. The oxygen level in the water tanks was 7.1 ± 0.6. During the 8-week experiment, the shrimp were fed a diet three times daily at 9:00 a.m., 2:00 p.m., and 6:00 p.m., allowing them to eat ad libitum under a natural photoperiod. The experimental design is reported in Table 1. After 3 weeks of feeding with a dietary 40%, shrimp was fasted for 1 week and refed in weeks 5–8 with various protein restriction schedules. We selected week 4 as we wanted to make sure the shrimp adapted to feeding and that they had enough time to compensate for the fasting. Therefore, week 4 was selected for fasting. The experimental groups were Control (40%), 7P40 (7 weeks feeding with dietary 40% protein and 1-week fasting), 6P40 (6 weeks feeding with dietary 40% protein, 1 week 35% protein, and 1-week fasting), 5P40 (5 weeks feeding with dietary 40% protein, 2 weeks 35% protein, and 1-week fasting), 4P40 (4 weeks feeding with dietary 40% protein, 3 weeks 35% protein, and 1-week fasting), and 3P40 (3 weeks feeding with dietary 40% protein, 4 weeks 35% protein, and 1-week fasting). We had a negative control: feeding shrimp with a diet of 35% for 8 weeks (Table 1).

### 2.3. Experimental Diets

Two experimental diets (dietary 40% and 35% protein) were prepared in this study to test protein restriction during the refeeding phase after fasting. The protein levels of 40% were selected based on previous studies in protein restriction and compensatory growth shrimp [12–17, 19]. We selected 35% as well to test our hypothesis. The lipid and energy contents were the same among diets (90 (g/kg) lipid and 18.5 (kJ/g)). The original ingredients were purchased from local markets. The main ingredients of this diet were fish meal, shrimp meal, squid meal, soybean meal, wheat flour and gluten, fish oil, soy oil, and lecithin. Feed manufacturing followed previously validated protocols, in which dietary ingredients were dried, mixed, and pressed pelleted [22]. We utilized a meat grinder (Electrokar EC-1, Tehran, Iran) to achieve the desired pellet size for this experiment. To reduce the moisture content of the pellets to below 100 g/kg, we spread them on a tray and placed them in an oven at 45°C for 24–48 h. Once dried, the pellets were packaged in nylon bags and stored at 4°C for subsequent analysis and shrimp feeding.  Table 2 shows the formulation and chemical composition of the experimental diets containing different protein levels.

### 2.4. Growth Indices and Analytical Methods

Following the experiment, all shrimp underwent a 24-h fasting period before being anesthetized in cold ice water. The formulas used to assess growth and feeding performance can be found in the footnote of Table 3. For the biochemical composition analysis, nine shrimp from each treatment group were selected and stored at −20°C. The proximate composition of the shrimp was analyzed according to AOAC standard methods [24]. The dry matter content (method 924.10) was determined gravimetrically after homogenized samples were dried in an oven at 105°C for 24 h (AMB50; ADAM, Milton Keynes, UK). Crude protein content (method 920.39) was assessed using the Kjeldahl method, employing an automatic Kjeldahl system (BÜCHI, Auto-Kjeldahl K-370; Switzerland), with nitrogen converted to protein using a factor of 6.25. Crude lipid content (method 920.39) was measured through ether extraction using a Soxhlet apparatus (Barnstead/Electrothermal, UK). Finally, the ash content (method 923.03) was determined by incinerating samples in a muffle furnace (Finetech, Shin Saeng Scientific, Paju-si, Gyeonggi-do, South Korea) at 550°C for 6 h.

### 2.5. Sample Collection

Nine shrimp were sampled from each tank to measure hemolymph biochemistry, immune system, antioxidant parameters, and serological enzymes. The hemolymph was collected directly from the cardiac sinus of shrimp using sterile syringes and transferred to centrifugal tubes on ice. Next, hemolymph tubes were put for 4 h in the fridge (4°C) to ensure that the hemolymph was clotted. Following centrifugation at 1500 rpm for 10 min at 4°C, the serum was isolated from the hemolymph. Subsequently, the hepatopancreas was dissected and rinsed in ice-cold phosphate buffer (100 mM potassium phosphate buffer (pH = 7.4), 100 mM KCl, 1 mM EDTA). After washing, the tissue was immediately frozen in liquid nitrogen and stored at −80°C for subsequent analyses [21].

### 2.6. Digestive Enzymes

Nine frozen intestine samples per group were thawed at 4°C, homogenized in 30 vol (v/w) of cold buffer (50 mM mannitol, 2 mM Tris-HCl buffer, pH 7.0), and centrifuged for 3 min at 3300 × *g*. The supernatants were then separated to analyze digestive enzyme activity. Lipase activity was assayed by using *p*-nitrophenyl myristate as a substrate. One unit of lipase activity was defined as 1 μmol of *n*-nitrophenol released per minute in 405 nm wavelength [25]. The amylase activity was measured following the methodology proposed by Bernfeld [26], using starch as a substrate [26]. One unit was calculated as the quantity of enzyme that released 1 µmol of maltose in 1 min. Specific activity (*U*) was expressed as amylase activity = (maltose released (µmol))/(3 × mg protein) [2]. Digestive enzyme activities were expressed as total activity per gram of the intestine tissue using a UV/visible spectrophotometer (Shimadzu UV/visible Spectrophotometer, Kyoto, Japan). Total protease activity was assayed using casein as the reaction substrate, following the method described earlier [27]. The protein content in the samples was quantified using the Bradford method [28].

### 2.7. Antioxidant Enzyme, Hemolymph Biochemistry, and Immune Parameters

Hepatopancreatic antioxidant enzyme activities were determined as described previously [29]. Briefly, frozen hepatopancreases were homogenized in 100 mM ice-cold phosphate buffer (9 ml buffer/g tissue) using a homogenizer for 30–45 s. The homogenate was centrifuged at 12,000 × *g* for 30 min at 4°C. Supernatants were collected and stored at −80°C for subsequent analyses. Lipid peroxidation was assessed using the thiobarbituric acid reactive substances (TBARSs) assay, modified from Reilly and Aust [30]. Briefly, samples were treated with trichloroacetic acid, butylated hydroxytoluene, and hydrochloric acid, followed by heating and centrifugation. Malondialdehyde (MDA) levels, indicative of lipid peroxidation, were determined by measuring absorbance and comparing it to a standard curve. Catalase activity was measured spectrophotometrically at 240 nm, according to Aebi [31]. The assay involved monitoring the decomposition of hydrogen peroxide (H_2_O_2_) in the presence of the enzyme extract. Briefly, 100 microfluids of supernatant were added to an assay mixture containing 1.9 ml of 0.05 M potassium phosphate and 1 ml of 0.059-M (30% volume/volume) H_2_O_2_ (pH 7), and absorbance was recorded at 240 nm for 3 min [21]. The soluble protein of extracts was measured according to the Bradford method [28] by use of bovine serum albumin as standard. The activity of superoxide dismutase (SOD) was measured following the method outlined by Kono [32]. For the assay, 500 μl of homogenate or serum samples were combined with 1300 μl of a reaction solution containing carbonate buffer (pH: 10.2), 500 μl of nitro blue tetrazolium (60 μM), 100 μl of Triton X-100 (0.6%), and 100 μl of hydroxylamine hydrochloride (20 mM, pH: 6.0). The optical density was recorded at 540 nm over a period of 5 min at room temperature to assess SOD activity, as per Kono's protocol [32].

To assess total protein, triglyceride, cholesterol, glucose, alkaline phosphatase (ALP) activity, aspartate aminotransferase (AST), alanine aminotransferase (ALT), lactate dehydrogenase (LDH), glutathione Peroxidase (GPx), and acid phosphatase (ACP) in hemolymph and hepatopancreases samples, diagnostic kits (Nanjing Jiancheng Bioengineering Institute, Nanjing, China) with an automatic biochemistry autoanalyzer was used (Technicon RA-1000, USA). The activity of phenol oxidase was assessed by quantifying the dopachrome generated from l-dihydroxyphenylalanine (l-DOPA) at a wavelength of 490 nm, utilizing an ELISA reader (BIORAD, USA), as described by Hernández-López, Gollas-Galván, and Vargas-Albores [33]. To determine serum lysozyme, Gram-positive bacteria sensitive to the lysozyme enzyme method were used (*Micrococcus lysodeikticus*) as substrate [34].

### 2.8. Evaluation of the Relative Expression of Genes

The expression of insulin-like growth factor 1 (IGF1), prophenoloxidase (ProPO), lysozyme, penaeidin-3a, and the housekeeping gene beta-actin in the hepatopancreas was assessed (Table 4), nine frozen tissue samples per group were homogenized in lysis buffer. Total ribonucleic acid (RNA) was isolated using a *High Purity RNA Isolation Kit* (Roche, Germany). RNA quality was assessed by 1% agarose gel electrophoresis, and its concentration and purity were determined using a NanoDrop ND-1000 spectrophotometer. Samples with 260/280 nm absorbance ratios between 1.86 and 2.00 were selected for further analysis. cDNA was synthesized from 1 µg of purified RNA using a 2-step kit (CinnaGen, Iran) and random hexamer primers. Primers were designed using the primer3 online software based on the gene sequences. Beta-actin served as the housekeeping gene for normalization [29]. For the quantitative real-time PCR (RT-PCR) analysis, we utilized a quantitative thermal cycler (Rotorgen2000, Corbett Research, Australia) and employed the RealQ Plus 2x Master Mix Green (Amplicon, Denmark). Each sample was analyzed in triplicate to ensure accuracy and reproducibility. The RT-PCR cycling conditions were set as follows: an initial denaturation at 95°C for 15 min, followed by 40 amplification cycles consisting of 95°C for 20 s, 58°C for 30 s, and 72°C for 30 s. A melting curve analysis was performed to assess the specificity of the amplified products by gradually increasing the temperature from 55 to 95°C at a rate of 0.5°C per 30 s. PCR amplification was conducted at identical temperatures for all target genes. The specificity of PCR products was verified by agarose gel electrophoresis. PCR efficiency was determined by performing a five-point serial dilution with three technical replicates. Three independent biological replicates, each with three technical replicates, were performed to calculate the average Ct values [29]. The expression analysis data were analyzed using the 2^−*ΔΔ*CT^ method [35].

### 2.9. Statistical Analysis

A completely randomized design with six treatments and three replicates was employed. Normality and homogeneity of variances were assessed using the Shapiro–Wilk and Levene's tests, respectively. Data were analyzed using one-way ANOVA with SPSS Version 22.0 for Windows. Tukey test was used to compare growth performance, body composition, hemolymph parameters, digestive enzymes, immune system, antioxidant activities, serological enzymes, and gene expression between the seven treatments. Additionally, correlation (Pearson's correlation test) was used to measure the relationship between measured physiological factors. Significance levels were set at *p* < 0.05 and *p* < 0.01.

## 3. Results

### 3.1. Growth Performance and Body Composition

**Figure 1:**
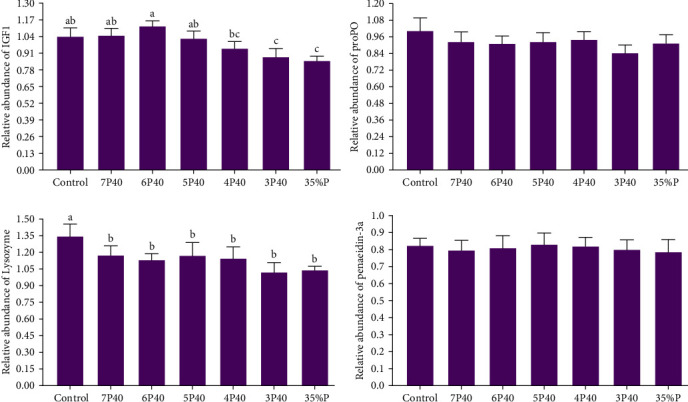
Relative expression of IGF1, lysozyme, ProPo, and penaeidin3a mRNA of the hepatopancreas of whiteleg shrimp after 8 weeks of feeding experimental diets under protein restriction and fasting schedules. Significant differences in treatment are indicated by letters a, b, and c based on Tukey's range tests (*p* < 0.05).

The growth performance data are presented in Table 3. The results indicated that the protein restitution during refeeding affected weight gain, specific growth rate, feed efficiency, daily feed intake, and protein productive value. The shrimp fed Control (10.22 g), 7P40 (9.37 g), and 6P40 (9.27 g) had higher values of weight gain than others (*p* < 0.05). Daily feed intake (5.46) in Control was significantly lower than that of those fed a dietary intake of 35% protein. We could see if shrimp fed dietary 40% protein during refeeding, and even with 1 week of fasting out of an 8-week period, there is still no significant difference among groups (10.22 vs. 9.37 g). In the current study, protein restitution and fasting did not affect protein efficiency ratio (PER), lipid efficiency ratio (LER), or survival rate. The survival rate of the shrimp was 100% for all treatments, showing that shrimp can tolerate fasting or protein restriction without problems. In the present study, protein restriction during the realimentation phase affected gene expressions of IgF1 (Figure 1). Those exposed to 3P40 (0.88) and 35%P (0.85) treatments had lower values of IgF1 than other groups but not 4P40 (*p* < 0.05).

The current study demonstrated that there was no significant change in ash content, which can be due to the fact that the ash contents in diets were not significantly different (Table 5). There was a clear trend in the body's protein content, and for those fed dietary 40% more weeks, protein levels in the body were higher as well. Accordingly, those fed Control (146.30 g/kg) and 7P40 (143.90 g/kg) had higher values than other groups (*p* < 0.05). Crude lipid showed approximately the same trend, and lipid in Control (50.10 g/kg) was higher than 5P40 (45.00 g/kg), 4P40 (32.90 g/kg), 3P40 (36.01 g/kg), and 35%P groups (40.40 g/kg). Moisture content in the shrimp body in 4P40 (775.40 g/kg) and 3P40 (777.30 g/kg) groups was higher than others but not 35%P treatment (*p* < 0.05). It shows whiteleg shrimp fed 35% protein diets more, had more moisture, and less protein and lipid in their body compared to those who ate more weeks with the dietary 40% protein.

### 3.2. Digestive Enzymes

**Figure 2:**
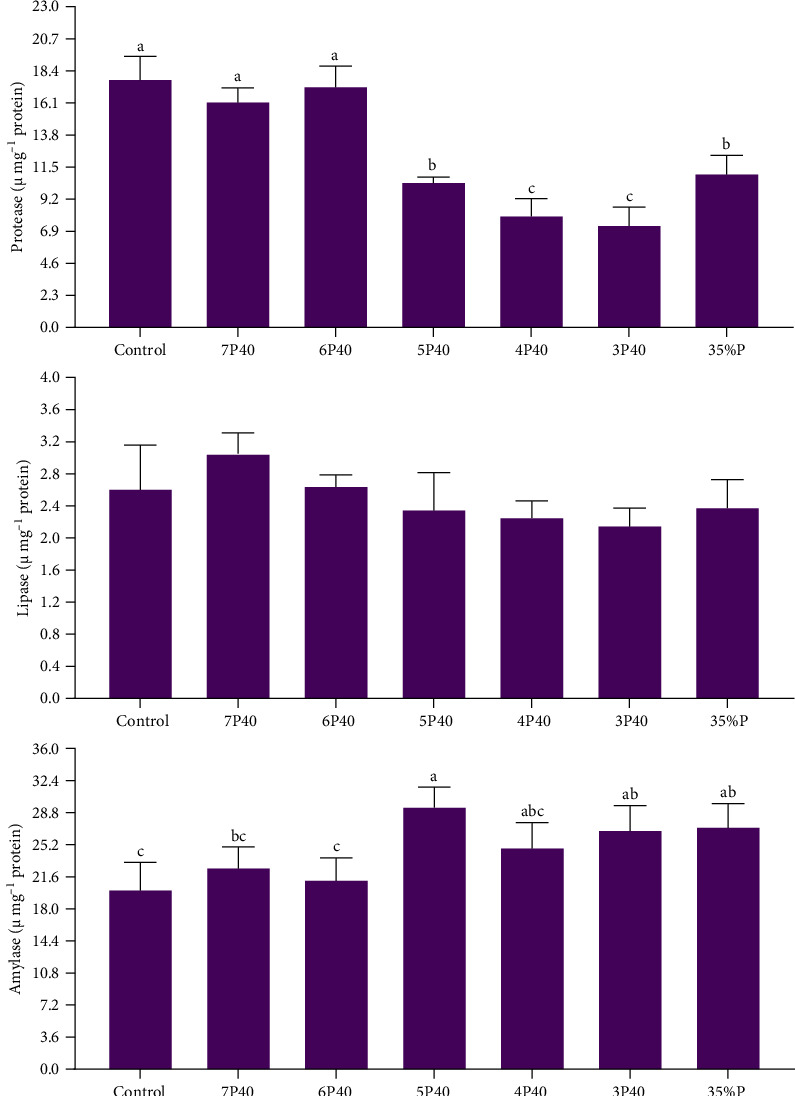
Digestive enzyme activity in the intestine of whiteleg shrimp after 8 weeks of feeding experimental diets under protein restriction and fasting schedules. Significant differences in treatment are indicated by letters a, b, and c based on Tukey's range tests (*p* < 0.05).

Figure 2 shows the result of the digestive enzyme in whiteleg shrimp under protein restriction and fasting treatments. Protease activity (U/mg protein) followed the amount of fed protein so that those experienced Control (17.74), 7P40 (16.13), and 6P40 (17.22) schedules had significantly higher values than others (*p* < 0.05). There was no significant difference in lipase activity among groups. Amylase activity in treatments who eat less protein (higher carbohydrate) was higher, so the activity of this enzyme in 5P40 (29.34), 3P40 (26.73), and 35%P (27.10) groups was greater than in Control (20.04) and 6P40 (21.12) treatments (*p* < 0.05). In general, 1 week of fasting and then 4 weeks of refeeding did not affect digestive enzymes.

### 3.3. Chemical Parameters of the Hemolymph

**Figure 3:**
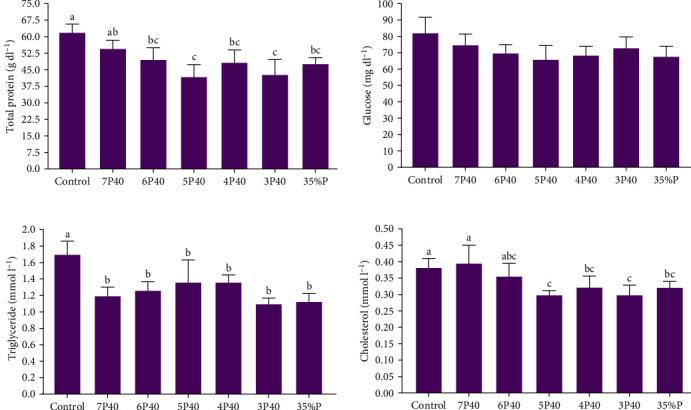
Serum hemolymph chemical parameters of whiteleg shrimp after 8 weeks of feeding experimental diets under protein restriction and fasting schedules. Significant differences in treatment are indicated by letters a, b, and c based on Tukey's range tests (*p* < 0.05).

Figure 3 presents the total protein, triglyceride, cholesterol, and glucose levels across the different treatments. The present study did not show significant differences in glucose among treatments. The total protein level was affected, and the Control (2.19 g/dl) had significantly higher values than the other groups but not 7P40 (*p* < 0.05) (Figure 3). The triglyceride level was depleted in experimental groups, and Control (1.69 mmol l^−1^) had significantly the highest level (*p* < 0.05). Cholesterol levels followed the same trend, and this parameter was higher in the Control (0.38) and 7P40 (0.39) groups than in other but not 6P40 treatment (*p* < 0.05).

### 3.4. Immune System, Antioxidant Activities, and Relative Gene Expression

**Figure 4:**
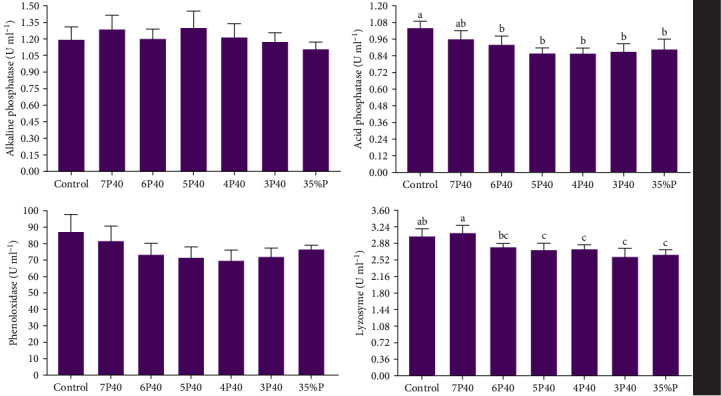
Hepatopancreas immune system parameters of whiteleg shrimp after 8 weeks of feeding experimental diets under protein restriction and fasting schedules. Significant differences in treatment are indicated by letters a, b, and c based on Tukey's range tests (*p* < 0.05).

The immune system parameters, including ALP, ACP, lysozyme, and phenoloxidase, were reported in Figure 4. ALP and phenoloxidase were not affected by protein restriction and fasting-refeeding schedules. The Control group had a higher value of ACP (1.04 U/ml) than others but not 7P40 (*p* < 0.05). The lysozyme also showed the same trend, so this parameter in Control (3.03) and 7P40 (3.10) was significantly greater than other treatments but not 6P40 (*p* < 0.05). These results showed that whiteleg shrimp fed more weeks with a dietary 35% protein immunity system was impaired.

**Figure 5:**
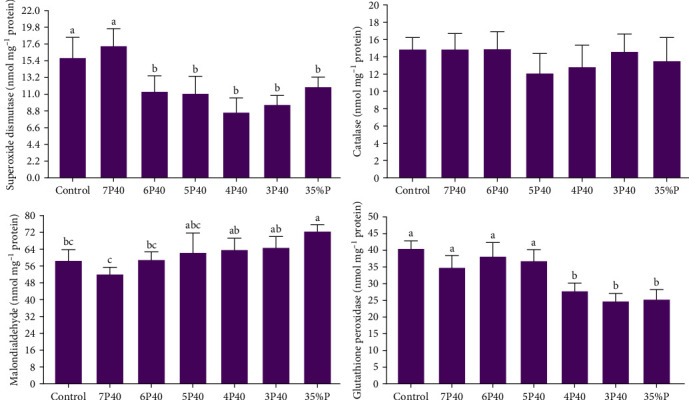
Hepatopancreas antioxidant activities of whiteleg shrimp after 8 weeks of feeding experimental diets under protein restriction and fasting schedules. Significant differences in treatment are indicated by letters a, b, and c based on Tukey's range tests (*p* < 0.05).

The current study demonstrated that the three evaluated parameters of the antioxidant system—MDA, GPx, and SOD (but not catalase)—along with the immune system were adversely impacted by protein restriction and fasting schedules (*p*  < 0.05) (Figure 5). ANOVA analysis indicated that Control and 7P40 treatments had a higher value of SOD (15.71 and 17.25 nmol/mg protein) than other groups (*p* < 0.05) (Figure 5). A lower value of MDA in those who were put to Control (58.31 nmol/mg protein), 7P40 (51.83 nmol/mg protein), and 6P40 (58.76 nmol/mg protein) schedules than 35%P group were observed. While catalase was not changed, GPx in 4P40 (27.67 nmol/mg protein), 3P40 (24.60 nmol/mg protein), and 35%P (25.14 nmol/mg protein) groups were lower than other treatments (*p* < 0.05). Taken together, protein restriction during the realimentation phase impaired the immune system and antioxidant activity.

In the present study, protein restriction during the realimentation phase affected gene expressions of lysozyme (Figure 1). The gene expression of lysozyme in Control (1.34) was significantly higher than others (*p* < 0.05).

### 3.5. Serological Enzymes

**Figure 6:**
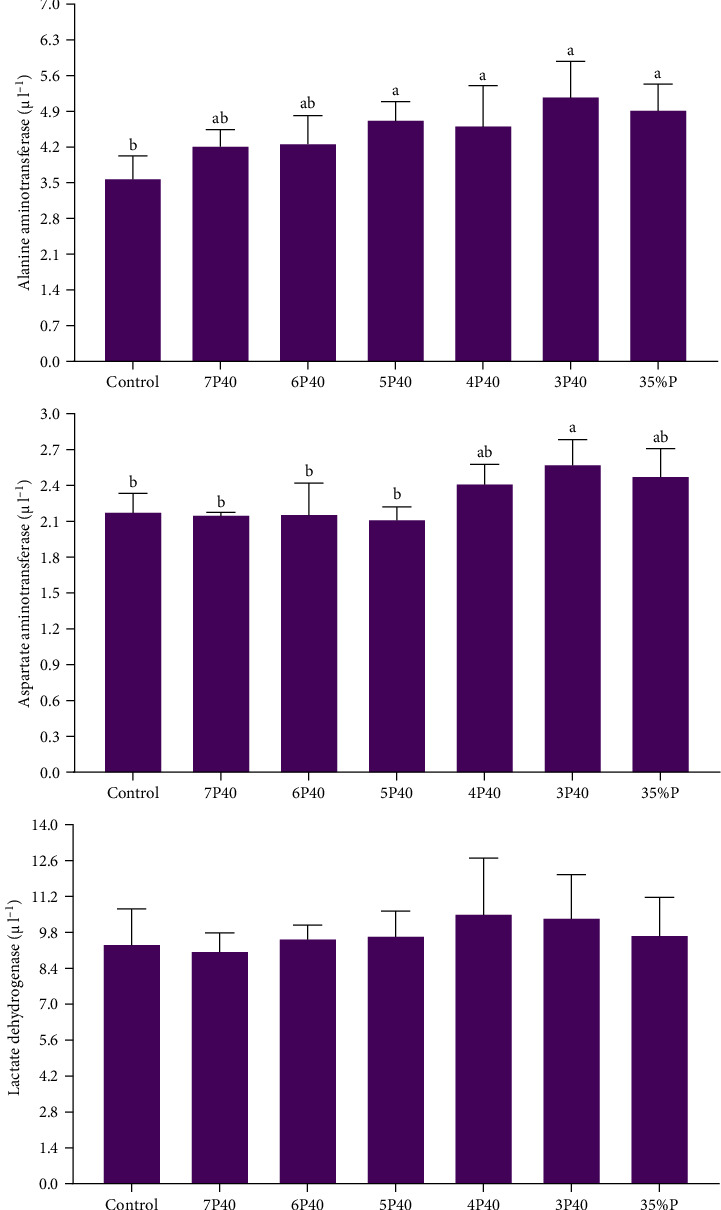
Serological enzyme parameters in the serum of whiteleg shrimp after 8 weeks of feeding experimental diets under protein restriction and fasting schedules. Significant differences in treatment are indicated by letters a, b, and c based on Tukey's range tests (*p* < 0.05).

Figure 6 indicates the change of serological enzymes (ALT, AST, and LDH) in whiteleg shrimp that experienced various protein restrictions and fasting/refeeding schedules. While LDH did not differ among groups, Control (3.56) had a lower value of ALT than other treatments but not 7P40 and 6P40 (*p* < 0.05). AST showed the same trend, and this parameter in Control (2.17), 7P40 (2.14), 6P40 (2.15), and 5P40 (2.11) were less than 3P40 (*p* < 0.05). This result shows that 35% protein has caused some health issues in shrimps.

### 3.6. Correlation Between Measured Parameters

**Figure 7:**
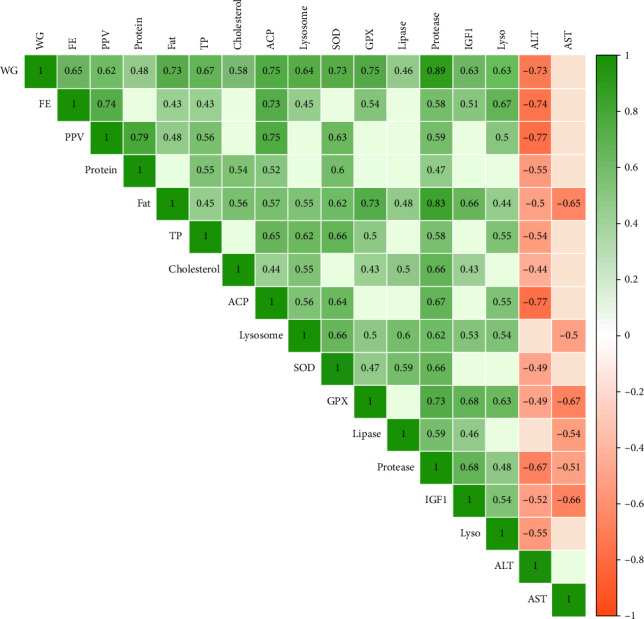
Correlation analysis of measured parameters after 8 weeks of feeding experimental diets under protein restriction and fasting schedules in whiteleg shrimp. Significant differences in treatment are in 0.05 and 0.01. Thresholds for 0.05 and 0.01 are 43.4 and 55.1, respectively (*p* < 0.05).

In the present study, protein restriction and fasting schedule caused a significant correlation among parameters (Figure 7). For example, the weight gain had positive significant correlations (the numbers in parenthesis show correlation coefficient R) with feed efficiency (65%), protein productive value (62%), body protein (48%), body lipid (73%), total protein (67%), cholesterol (58%), ACP (75%), lysozyme (64%), SOD (73%), GPx (75%), lipase (46%), protease (89%), IgF1 (63%), and lysozyme gene (63%). Also, weight gain had a negative correlation with ALT (−73%). Further, feed efficiency had a correlation with protein productive value (74%), body lipid (43%), total protein (43%), ACP (73%), lysozyme (45%), GPx (54%), protease (58%), IgF1 (51%), and lysozyme gene (67%). The Protein in the body had a strong connection with protein productive value (79%), total protein (54%), cholesterol (54%), ACP (52%), SOD (60%), and protease (47%). Similarly, lipid content had a strong positive connection with all parameters, which are shown in green in Figure 7. Total protein, which is an indicator of fish health and protein metabolism, has a strong connection with growth performance parameters, immune parameters, the antioxidant system, and related genes. Immune system parameters had a strong correlation with each other, as did growth performance and antioxidant parameters. SOD also has a strong correlation with GPx (47%), lipase (59%), protease (66%), growth, and immunity. IGF1 also has a positive correlation with growth performance, body lipid (66%), cholesterol (43%), lysozyme (53%), GPx (68%), lipase (46%), and protease (68%). Finally, ALT had an almost negative correlation with all the abovementioned parameters and to a lesser extent, AST showed the same trend. All in all, the results indicate that when whiteleg shrimp could grow, it had a better health status, and the whole body worked to shift energy toward growth.

## 4. Discussion

### 4.1. Growth Performance, IGF1 Expression, and Body Composition

Feeding strategies have been used for the long term to improve feed efficiency, growth, and water quality. While much aquaculture research has focused on ingredients, additives, and diet formulation, feeding strategies can improve growth and feed efficiency. Several approaches are currently being tested for compensatory growth and protein restriction. The present study indicated that we cannot benefit from both protein restriction and fasting strategies. On week fasting in week 4, impaired growth performance and shrimp could not compensate for growth with feeding dietary 35% protein. However, shrimp could grow the same as Control with feeding every other week dietary 35% and 40% (unpublished data). Further, comparing the Control and 7P40 groups showed that fasting alone does not impair growth if shrimp are fed with dietary 40% in refeeding time. The current study showed shrimp under both protein restriction and fasting schedule can grow well if maximum they are fed only 1 week of dietary 35% protein out of 8 weeks (6P40 group). Finally, our study reported that 35% protein is not enough for whiteleg shrimp, and 40% protein should be provided for optimum growth and health.

Other shrimp studies showed that this species can tolerate up to 7 days of fasting without any problem. They showed a 100% survival rate and only a nonsignificant 3.2% loss of weight [11, 13, 14]. Further, crustaceans tolerate, on average, these 5–7 days of fasting during molting, and it is a normal part of their life cycle. All the other physiological conditions, such as hemolymph biochemistry, digestive enzyme activities, and the immune system, get back to normal baseline after a maximum of 1 week after fasting [13]. Therefore, we selected 1 week of fasting to test how it interacts with protein restriction. Shrimp in the current study had 4 weeks of refeeding after fasting; it is more likely all the changes from this study, which were sampled in week 8th, have been due to protein restriction and not fasting. The density of farmed shrimp and the time of fasting are also important, which was not the case for our study as all treatments (not positive and negative control) experienced 1 week of fasting with the same number of shrimps per tank. Similar to previous studies [13, 14], our data showed that 7 days of fasting did not impair growth performance and physiological metabolisms when shrimp were fed optimum dietary protein (40%, 7P40).

Despite the remarkable effects on feed cost, improved feed efficiency, growth, and water quality, protein restriction has not been well studied enough. Our previous studies in Siberian sturgeon (*Acipenser baerii*) indicated that feeding with 30% and 40% dietary protein every other day and every other week did not significantly decrease growth performance and feed efficiency compared with a control group [22]. Similar to Siberian sturgeon, rainbow trout (*Oncorhynchus mykiss*) from our project showed the same results in terms of growth and feed efficiency, feeding every other week with dietary 35% and 45% proteins [36]. Unlike Siberian sturgeon and our data, protein restriction did not improve feed efficiency in rainbow trout. In another investigation of rainbow trout, protein restriction also worked well so that 3 out of 9 weeks, feeding restricted protein (30% compared to Control 40%) did not suppress growth performance [37]. The researcher also tested daily schedules, and when a Japanese flounder (*Paralichthys olivaceus*) was fed 18 days with a diet of 40% protein, and then 30 days with a control diet (50%), the growth in this group did not differ from the control [38]. In these studies, 3 and 5 weeks of feeding with lower and optimum protein levels, respectively, impaired growth performance, showing a threshold that exists for each fish species to tolerate lower protein levels in a protein restriction schedule. For whiteleg shrimp, it is impossible to introduce this threshold as fasting was also a driver in the results obtained. A decrease in protein deficiency occurred in other studies in whiteleg shrimp, yellow catfish (*Pelteobagrus fulvidraco*), rainbow trout, Siberian sturgeon, and barramundi (*Lates calcarifer*) [22, 36, 39, 40], which is unlike our study.

In the limited studies in protein restriction in crustaceans, Chinese shrimp (*Fenneropenaeus chinensis*) (1.3 g) were fed with restricted protein for weeks one and two (29% protein) and then fed with dietary control (44% protein) for four more weeks; the growth performance, feed efficiency, and PER did not differ than the control group [18]. Whiteleg shrimp were exposed to 70% feed restriction during the postlarval stage over 3 days and then farmed for various dietary proteins (30%, 37%, and 43%) for 70 days [41]. Lage et al. [41] reported that no significant difference was observed among groups in terms of growth and feed efficiency. When whiteleg shrimp were fed 3 out of 6 weeks with restricted dietary protein (36%), they showed no significant growth performance compared with control (43% protein) [19]. However, four out of 6 weeks of protein restriction were too much in this study. Crayfish (*Cherax cainii*) were fed for 2 weeks with a diet of 17% protein and then routine dietary feeding (36% protein). The results indicated that the growth performances were decreased with a modified diversity of intestinal microbiota [42]. Most protein restriction studies reduce protein levels by a maximum of 10%, but they reduced it in this study by more than 20%, possibly reducing growth performance. All these studies in crustaceans showed that protein restriction could be a promising approach to optimize growth and feed efficiency, which was not observed in our study.

Also, improved feed efficiency with restriction feeding and fasting/refeeding schedules due to improved digestive enzymes and/or metabolism and improved water quality were observed in shrimps [6, 7, 13]. In our study, fasting could not improve feed efficiency (Control vs. 7P40). Adding protein restriction to the refeeding schedule again could not improve feed efficiency, but there was no significant difference between Control and up to the 4P40 group that fed dietary 40% protein only in the last week. 35%P group also had lower feed efficiency, and growth shows clearly that 35% protein is not for shrimp farmed in our experimental conditions. Even if improved feed efficiency could not happen, protein restriction is beneficial as protein-restricted diets are cheaper than Control. Further, animals with less protein degradation are more likely to have better feed efficiency [43, 44], which is a possible reason for catch-up growth. The relation between protein degradation and restriction should be much more studied.

In shrimp that were not sufficiently fed protein, one strategy to meet the requirement is to enhance the feeding rate (hyperphagic response), which has been shown well previously [40, 45]. This was also clearly observed in our data, and the 35%P group had higher daily feed intake than the control group. Perhaps the 35%P group tried to apply various behavioral and physiological mechanisms to maximize protein efficiency, but they could not, as the growth was significantly lower in this group. It also shows that shrimp, like other aquatic species, required a balanced amino acid diet irrespective of dietary protein level to some extent (not very low). However, there is a threshold for feed intake, and no further shrimp performance is gained after the threshold. The present study indicated that both diets met the amino acids requirement of whiteleg shrimp. Digestibility of these two diets (that is a driver of amino acid availability) was also at the standard level when we compared these diets with other digestibility studies in this species [46, 47]. The main issue could potentially be 36% wheat meal, which is in the standard range for whiteleg shrimp. All this information is further evidence that 35% protein in our study is standard.

Further, we investigate the pattern of gene expressions of IGF1 as a central gene in metabolism. IGF1 stimulates the growth of all cell types and is responsible for sending signals to cells about the availability of nutrients to go toward growth and inhibit cell apoptosis [48]. IGF-1 directly stimulates the mTOR signaling pathway [49] and, along with amino acids, is the main stimulator. Long-term protein restriction inhibited the mTOR signaling pathway, declined muscle protein synthesis, and impaired shrimp growth performance [19]. Further, the activation of the mTOR signaling pathway in treatments with increased growth performance and feed efficiency was observed [16]. In our study, lower IGF1 in 3P40 and 35%P shrimps, along with reduced growth and protease in these groups, are a well-matched puzzle. In other studies on whiteleg shrimp, groups fed more with dietary high protein had higher growth performance, and more than 10 genes involved in amino acid catabolism, gluconeogenesis, and glucose metabolism were enriched [41].

Several factors, such as age, size, gender, water quality, and environmental parameters, can shape crustaceans' muscle composition. Among these factors, diet is the main driver of changes. The present study showed that when whiteleg shrimp were fed more protein in diets, protein, and lipid content in the body increased. These results are unlike other studies that showed protein restriction did not impair protein levels in the body even every other week [22, 36]. In rainbow trout, higher and lower values of protein and lipid contents in the protein-restricted group that had the same growth in comparison with the Control were observed [36]. In our study, growth could not be compensated after fasting, and protein and lipid could not either. In yellow catfish, however, no significant difference in protein levels and even the whole-body EAA or NEAA concentrations was observed [40]. However, these results are similar to those of Chinese shrimp, so there was a direct relation between protein and fat in the body and dietary protein levels [18]. In crayfish with scheduled protein restriction that was mentioned in the last section, the protein level was maintained while the growth was decreased [42].

### 4.2. Digestive Enzymes

Digestive enzyme activity can be an indicator of the digestion of nutrients, and indirectly, animals with higher digestive enzymes can potentially have higher growth and feed efficiency. This research is about protein restriction, and unsurprisingly, protein and amylase were changed in this study as diets had various protein and carbohydrate levels. Short-term fasting cycles in our study were probably safer not to compromise enzyme production. Studies showed that protein is the key nutrient during refeeding as protease activities and also trypsin and chymotrypsin activities in shrimp subjected to fasting were remarkably increased after refeeding [13, 50]. Also, lipase and amylase, to a lesser extent, were elevated in these studies [9] during refeeding in whiteleg shrimp to balance the energy mechanism through lipids and carbohydrates.

Eating less high-protein diets has been a reason why the growth and digestive enzymes from 5P40 to 35%P groups were lower than Control, as enough protein was not provided. Treatments were fed for more weeks with a diet of 40%; they had higher protease and lower amylases. However, lipase remained constant among groups in our data. Zhao et al. [19] indicated that in restricted protein treatments (3 out of 6 weeks), there was no difference in growth, digestive enzyme activities, and gene expression levels, and the crude protein content of shrimp muscle did not differ either. In groups fed, 4 out of 6 weeks of restricted protein diets, growth decreased, but not digestive enzyme activities and gene expression levels. Whiteleg shrimp were exposed to 70% feed restriction during the postlarval stage over 3 days and then farmed for various dietary proteins (30%, 37%, and 43%) for 70 days [41]. Lage et al. [41] reported no significant difference among groups in terms of gene expression of lipase and protease, but amylase was higher in those fed dietary 43% protein compared to those fed dietary 36% protein.

There are several studies that indicated that when shrimps were fed with a protein diet that was not optimum, digestive enzymes were also changed. Trypsin activity (is a protease) and lipase in whiteleg shrimp increased with the increasing dietary protein level (the highest level was 34%), but amylase activity showed a converse trend [51]. While 43% was introduced as the optimum protein level, protease and lipase were elevated by the optimum protein level, and amylase decreased in this species [52]. Trypsin, lipase, and amylase were higher in dietary 35% compared to 30% in whiteleg shrimp [53]. Greasyback shrimp (*Metapenaeus ensis*) is similar to whiteleg shrimp in terms of physiology, and the optimum protein level is 39%. Trypsin and pepsin increased, amylase decreased, and lipase remained relatively constant at this optimum level compared to other treatments [54].

Generally, increased digestive enzyme activity can be connected to improved digestion and absorption of nutrients that eventually improve growth performance and feed efficiency. Our data and others show that the more protein they eat up to the optimum level, the higher their digestive enzyme activities are. While whiteleg shrimp in 4P70 were fed 1 week with normal protein, the digestive enzymes did not recover. This shows how nutritional history affects shrimp, and future studies should test this phenomenon. There was a clear trend between improved growth and feed efficiency and higher protease in the 6P40 group compared to other fasted and protein-restricted groups.

### 4.3. Biochemistry Parameters of the Hemolymph

Crustaceans have an open-vessel circulatory system, and hemolymph is a route for the interorgan transport of nutrients. Hemolymph is like blood for crustaceans, and measuring its biochemical parameters can show the physiological and immunological conditions of animals [55]. Glucose, total protein, cholesterol, and triglyceride in hemolymph in whiteleg shrimp got back less than 24 h of feeding to baseline after 5 days of fasting [12]. Therefore, it can be claimed that in our research, fasting did not influence the hemolymph biochemistry parameters, and most of the changes came from protein restriction. The present study did not reveal significant differences in glucose among treatments. However, cholesterol, triglyceride, and total protein were lower in those fed more low-protein diets (5P40, 4P40, 3P40, and 35%P) compared to Control. There is only one study regarding the effect of protein restriction on blood or hemolymph biochemistry parameters. Whiteleg shrimp were exposed to 70% feed restriction during the postlarval stage over 3 days and then farmed for various dietary proteins (30%, 37%, and 43%) for 70 days [41]. They found that no significant difference was observed among the group in terms of glucose, but triglycerides were higher in those fed dietary 43%. During refeeding or compensatory growth, high variability in energy reserve mobilization in shrimps occurred, and which nutrient is used during this period depends on the size of the animal, severity and time of feed restriction, available nutrients in feed, etc. [56]. Many more studies are required to connect the biochemistry parameters to the energy turnover during protein restriction in aquatic species.

When the quality or quantity of protein in a diet is not optimum, biochemistry parameters are reduced. For example, feeding whiteleg shrimp with optimum protein (42%) increased triglyceride compared with those fed 30%, while cholesterol remained at the same level [57]. In another study of this species, four different protein sources were tested, and cholesterol, feed consumption, feed efficiency, protein consumption, and PER were affected by the quality and quality of protein sources [58]. Similarly, there was an upward linear relation between total protein and cholesterol and protein levels in whiteleg shrimp diets up to the optimum level [59, 60]. Total protein, cholesterol, and triglyceride in those fed 30% did not differ from 35%, but growth was higher in shrimp fed dietary 35% protein [53]. All these studies are in line with our data, which shows that these parameters are directly affected by dietary protein. In conclusion, our study indicated that protein restriction can directly affect hemolymph biochemistry parameters.

### 4.4. Antioxidant Activities and Immune System

The immune system and antioxidant parameters are closely linked to each other in shrimp, and its activities are strongly related to proteins, and several immune and antioxidant components are protein based. The measuring of these parameters, such as lysosome, phenoloxidase, ACP, ALP, MDA, SOD, glutathione, GPx, and catalase, can be applied to monitor the health status of crustaceans. We can confidently claim that the changes have been due to protein restriction as Control and 7P40 in all immune and antioxidant parameters showed approximately the same level. Studies in this species reported even after fasting, 5–7 days is enough for immune parameters to return to their baseline values [14]. Also, parameters related to the antioxidant system got back to normal after 7 days of refeeding [14]. In our study, whiteleg shrimp were fed 4 weeks after fasting. In the current study, whiteleg shrimp had weaker immune parameters in those fed a dietary 35% protein, and the experiment finished up with 40% protein in most treatments. These results show that even after 1 week of feeding with a dietary 40% protein (4P40), shrimp still suffered from a lack of enough protein, eventually negatively affecting the immune and antioxidant systems. There are few studies that tested how the immune system can be changed via protein restriction. For example, studies on Siberian sturgeon and rainbow trout showed that those fed less protein had lower immune parameters [22, 36]. Also, lower hematological parameters in protein-restricted Acipenser were observed [61]. All these studies indicated that if even growth is not suppressed, the immune system can potentially decrease. Although more studies are required, growth and immunity usually go in the same direction. In other studies in crustaceans, lower lysozyme and ACP in aquatic species in the group fed “not optimum” protein were also observed [62, 63]. Similarly, SOD and catalase in muscle and hepatopancreas decreased, but total hemocyte count was increased by enhanced protein levels in whiteleg shrimp diets [51]. In greasyback shrimp, in optimum protein treatment in terms of growth, ALP, catalase, and SOD were the highest, and MDA was the lowest as well [54]. SOD and lysozyme in shrimp fed 35% protein were higher than those fed 30% [53]. When shrimp were reared in zero-water exchange bio floc-based intensive culture tanks for a period of 7 weeks, immune (total hemocyte count, phagocytic activity, antibacterial activity, and bacteriolytic activity), antioxidant systems (total antioxidant system, SOD activity, and glutathione level in the plasma and the hepatopancreas), and growth performance showed no significant differences among groups (25% and 35% protein) [64]. All these studies clearly indicated a positive trend between growth and the immune system of whiteleg shrimp, and accordingly, considering optimum protein in terms of the immune system and the antioxidant system is crucial.

While gene expression does not necessarily represent functional proteins and correlates to the amount of expressed protein, it still is a valuable assessment. In this study, for a better understanding of the role of protein restriction in the expression of genes related to immunity, lysozyme, ProPO, and penaedin3a were measured. Lysozymes, as the immune system component, degrade bacterial cell walls and remove infections in aquatic species of animals [65]. In the current research, all groups had a lower value of the lysozyme's gene compared to the Control, showing that both fasting in week 4th and protein retraction negatively affected the expression of this gene. This result is aligned with lysozyme levels, showing that protein restriction remarkably affects this protein and its genes. Other studies reported starvation also reduced the expression level of immune parameters and relative genes in crustaceans [66–68], which, of course, perhaps is not connected to our study (not lysozyme) as whiteleg shrimp refeed 4 weeks until the end of the experiment.

All in all, protein restriction negatively affects the immune system and antioxidant parameters. It is suggested that the immune system should also be considered when applying any feeding strategies. Perhaps animals respond well in terms of growth and feed efficiency but not immune systems.

### 4.5. Serological Enzymes

ALT, AST, and LDH are common parameters that have been used to monitor shrimp hepatopancreas health status. In the present study, fasting did not affect the group as there was no difference between Control and 7P40, which showed that fasting was not the main driver and that all changes were related to protein restriction. While LDH was not changed with protein restriction, ALT and AST were higher in those fed less protein. It shows that the body tries metabolizing more protein to compensate for not updating enough protein. There is no study regarding the effect of protein restriction on serological enzymes in aquatic animals. However, some studies showed that serological enzymes were elevated in animals fed dietary “not optimum” protein levels. Accordingly, AST and ALT increased, and LDH also decreased with feeding declined protein levels, which somehow aligns with our work [51]. In other studies, ALT and AST did not differ in shrimp fed dietary 30% and 35% protein [53]. In general, in most cases, groups with poor growth due to not feeding with an optimum protein had higher serological enzymes such as ALT, AST, and LDH [69–73]. On the other hand, the increased levels of ALT and AST suggested protein catabolism at high dietary protein levels, and the amino acid surplus from protein-rich diets cannot be directly stored in fish, and they might be deaminated and converted into energetic compounds [74]. The trends of elevated activities of both enzymes indicated that protein metabolism is more active in animals. We can see that feeding animals with both excessive or/and lesser dietary protein levels can be indicated by elevated serological enzymes. The exact mechanism of action for each case is unknown, but the clear output is that when the protein level is not at the “optimum level,” increased serological enzymes can occur. Much more studies are required on various species to arrive at a solid conclusion.

### 4.6. Correlation Between Measured Parameters

Examining the relationships between measured parameters in research can reveal significant insights into underlying biological mechanisms. This is much truer for studies that investigated growth with other parameters, such as the immune system and antioxidants. Crustaceans' bodies, like other animals, act like a system of biology, and any alteration from diet to stress can manipulate all physiological mechanisms to maintain homeostasis at an optimum level and eventually maximize growth and health. In the current data, there was a positive correlation between weight gain and protein level and fat in the body, hemolymph biochemistry, digestive enzymes, and immune and antioxidant parameters. This shows that all these parameters work together to ensure whiteleg shrimp have high growth or vice versa. Interestingly, higher levels of fat also showed higher levels of immune and antioxidant parameters. It can be speculated that in crustaceans, higher lipid levels in the body up to the threshold can show good health status. While there was no relation between cholesterol and total protein, these two parameters had a positive correlation with the immune and antioxidant systems. Protease (to a lesser extent lipase) had a positive correlation with growth, feed efficiency, and immune and antioxidant parameters, each of which is a piece of the system biology puzzle. These results are in line with previous studies on shrimps under different additives, such as montmorillonite [29], as was earlier reported in fish species [2, 75]. This question explores whether enhanced growth performance is a consequence of improved immune and antioxidant systems or if it serves as a driving factor for these improvements. Given the complexity of the relationship between health and growth, further research is necessary to clarify this issue.

ALT and, to a lesser extent, AST had a negative correlation with growth, immune system, digestive enzymes, and antioxidant activity, showing that these parameters can be used as an indicator of growth and health as was observed in earlier studies in aquatic species from our projects [76–78] and also other works [79]. While elevated ALT and AST do not mean animals suffer health issues directly, they still can be indicators of “nonoptimum conditions” in aquatic species.

## 5. Conclusions

Regarding growth performance and feed efficiency, there was no significant difference between those fed 6 weeks of dietary 40%, 1 week of dietary 35%, and 1 week of fasting compared to Control. One week of fasting itself did not negatively affect these groups when they fed dietary 40% (Control vs. 7P40). Also, 35% protein was not enough by far to provide maximum growth and health. While there was no significant difference in survival rate, the synergic effect of fasting and protein restriction (especially in 4P40 and 3P40 groups) suppressed immunity, hemolymph biochemistry, antioxidant system, and relative genes. Digestive enzymes also decreased in this group, and serological enzymes were higher. In conclusion, applying both fasting-refeeding and protein restriction is not recommended. Furthermore, testing protein restriction in longer term field-based experiments in whiteleg shrimp is suggested.

## Figures and Tables

**Table 1:** Experimental design for crosstalk between protein restriction and compensatory growth.

Treatments	Week 1 (%)	Week 2 (%)	Week 3 (%)	Week 4	Week 5 (%)	Week 6 (%)	Week 7 (%)	Week 8 (%)
Control	P40	P40	P40	P40%	P40	P40	P40	P40
7P40	P40	P40	P40	Fasting	P40	P40	P40	P40
6P40	P40	P40	P40	Fasting	P35	P40	P40	P40
5P40	P40	P40	P40	Fasting	P35	P35	P40	P40
4P40	P40	P40	P40	Fasting	P35	P35	P35	P40
3P40	P40	P40	P40	Fasting	P35	P35	P35	P35
Protein 35%	P35	P35	P35	P35%	P35	P35	P35	P35

*Note:* Fasting: 1 week without feeding.

Abbreviations: P35%, protein 35%; P40%, protein 40%.

**Table 2:** Experimental diets were used to feed whiteleg shrimp under protein restriction and compensatory growth.

Ingredients	Experimental diets (g/kg)		
	40% protein	35% protein	
Wheat, red W.	280	360.6	
Soybean meal −48%	258.5	256.7	
Gluten meal	80	10	
Fish oil	50.3	49.7	
Fish meal	255	250	
Squid meal	23.2	20	
Other ingredients^a^	53	53	
Proximate analysis (g/kg)			
Crude protein	401.0	352.0	
Crude lipid	90.8	91.1	
Ash	101.0	98.0	
Moisture	80.8	80.0	
NFE	345.2	390.9	
Fiber	68.0	62.0	
Gross energy (kJ g^−1^)^b^	18.6	18.4	
Amino acids (mg AA/g sample)			Requirements
Aspartic acid	24.70	23.39	—
Glutamic acid	106.96	86.73	—
Serine	16.69	14.19	—
Glycine	43.02	23.65	25
Histidine	14.42	10.60	8
Arginine	24.22	20.12	20
Threonine	15.01	23.04	12
Alanine	18.37	17.88	—
Proline	27.04	21.93	—
Tyrosine	5.69	8.83	—
Valine	22.03	19.31	14
Methionine	7.81	7.59	7
Isoleucine	23.25	17.56	11
Leucine	31.08	31.27	17
Phenylalanine	15.28	13.74	13
Lysine	25.87	29.26	18

^a^Other ingredients (g/kg): lecithin (10), shrimp meal (30), vitamin premix (5), mineral premix (5), DL-methionine (1), L-lysine HCl (1), and choline chloride (1).

^b^Gross energy values calculated based on 23.6 kJ g^−1^ proteins, 39.5 kJ g^−1^ fat and 17.2 kJ g^−1^ carbohydrates [23]. The proximate analysis of the diets was measured based on the standard AOAC method [24]. Nitrogen free extract NFE was calculated as, NFE = 100 – (crude protein+ lipid+ crude fiber+ ash), on a dry weight basis.

**Table 3:** Growth performance and feed utilization of whiteleg shrimp experienced protein restriction and compensatory growth for 8 weeks.

Parameter	Control (40%)	7P40	6P40	5P40	4P40	3P40	35%P
Initial weight (g)	0.28 ± 0.03	0.32 ± 0.03	0.31 ± 0.04	0.30 ± 0.03	0.30 ± 0.05	0.28 ± 0.02	0.29 ± 0.04
Final weight (g)	10.50 ± 0.70a	9.69 ± 0.64a	9.58 ± 0.37a	8.04 ± 1.07b	7.50 ± 0.55b	7.61 ± 0.53b	7.92 ± 0.37b
Weight gain (g)	10.22 ± 0.71a	9.37 ± 0.66a	9.27 ± 0.35a	7.74 ± 1.05b	7.20 ± 0.56b	7.33 ± 0.52b	7.64 ± 0.33b
Weight gain (%)	3636.16 ± 552.81	2954.74 ± 460.13	3011.85 ± 296.18	2578.88 ± 273.22	2451.16 ± 507.34	2658.87 ± 247.16	2682.56 ± 234.58
^†^SGR	6.45 ± 0.26a	6.09 ± 0.26ab	6.13 ± 0.17ab	5.87 ± 0.19b	5.76 ± 0.34b	5.92 ± 0.16b	5.94 ± 0.15b
^‡^Feed efficiency	62.31 ± 5.63a	57.95 ± 4.66ab	58.95 ± 4.49ab	55.73 ± 3.92ab	55.18 ± 5.84ab	53.46±3.69b	51.14 ± 3.88b
^§^DFI (%BW/day)	5.46 ± 0.47b	5.79 ± 0.49ab	5.70 ± 0.42ab	5.96 ± 0.40ab	6.02 ± 0.63 ab	6.23 ± 0.41ab	6.49 ± 0.52a
PER	1.60 ± 0.14	1.50 ± 0.12	1.56 ± 0.11	1.51 ± 0.11	1.53 ± 0.15	1.52 ± 0.10	1.45 ± 0.11
LER	7.00 ± 0.63	6.55 ± 0.53	6.66 ± 0.49	6.34 ± 0.44	6.29 ± 0.64	6.08 ± 0.40	5.83 ± 0.45
PPV (%)	31.93 ± 5.30a	29.16 ± 1.23ab	25.52 ± 3.06b	24.10 ± 0.86b	24.81 ± 2.43b	24.74 ± 1.92b	24.40 ± 1.22b
Survival rate (%)	100 ± 0.0	100 ± 0.0	100 ± 0.0	100 ± 0.0	100 ± 0.0	100 ± 0.0	100 ± 0.0

*Note:* Values are represented by means ± SDM of triplicate tanks; means without letter labels are not significantly different. According to Tukey's range tests, the letters a and b indicate significant differences in the treatments (*p* < 0.05).

Abbreviations: BW, body weight; DFI, daily feed intake; LER, lipid efficiency ratio; PER, protein efficiency ratio; PPV, productive protein value; SGR, specific growth rate.

^†^SGR (%BW/day) = (Ln final weight − ln Initial weight/days) × 100. Weight gain (g) = Final body weight − initial body weight. Weight gain (%) = 100 × (Final body weight − initial body weight)/(initial body weight).

^‡^Feed efficiency = Weight gain (g)/feed intake (g).

^§^DFI (%BW/day) = total feed fed (g) × 100/(average body weight (g) × days). PER = Shrimp wet weight gain (g)/protein intake (g crude protein). LER = Shrimp wet weight gain (g)/lipid intake (g crude lipid). PPV = ((shrimp protein gain (g crude protein))/protein intake (g crude protein)) × 100. Survival rate (%) = Number of shrimp in each group remaining on day 56/initial number of shrimp) × 100.

**Table 4:** Sequences of primers and amplification efficiencies for the expression of selected genes in whiteleg shrimp.

Gene	Sequences of primers	Accession number	Final product	Performance
Lysozyme	TGT TCC GAT CTG ATG TCC GCT GTT GTA AGC CAC CC	AY170126.2	121	98
Beta-actin	CCACGAGACCACCTACAAC AGCGAGGGCAGTGATTTC	AF300705.2	142	97
Penaeidin-3a	CACCCTTCGTGAGACCTTTG AATATCCCTTTCCCACGTGAC	Y14926	123	96
Igf1	CTCTGTACAGTCAGCCCAGC CACACCCAGTCAGTCCCAAG	XM02739466	220	92
ProPhenoloxidase	GAGATCGCAAGGGAGAACTG CGTCAGTGAAGTCGAGACCA	EF565469.1	243	93

**Table 5:** Proximate composition (wet weight) of whiteleg shrimp experienced protein restriction and compensatory growth for 8 weeks.

Proximate analysis (g/kg)	Treatments						
	Control	7P40	6P40	5P40	4P40	3P40	35%P
Crude protein	146.3 ± 9.3a	143.9 ± 7.3a	121.3 ± 7.7b	118.8 ± 7.9b	125.8 ± 7.0b	127.0 ± 8.5b	119.1 ± 9b
Crude lipid	50.1 ± 1.1a	47.7 ± 2.9ab	48.0 ± 3.5ab	45.0±3.0b	32.9 ± 1.2d	36.0 ± 1.7d	40.4 ± 2.3c
Ash	34.3 ± 3.1	33.5 ± 3.6	35.4 ± 5.9	32.3 ± 5.1	38.6 ± 3.6	35.7 ± 5.1	32.9 ± 1.8
Moisture	736.1 ± 15.4b	736.5 ± 15.7b	742.4 ± 15.6b	743.2 ± 14.7b	775.4 ± 10.0a	777.3 ± 15.0a	755.3 ± 10.2ab

*Note:* The proximate analysis of the body was measured based on the standard AOAC method [24]. Different lowercase letters indicate that the level of significance has been 0.05, which we mentioned in Section 2.

## Data Availability

Data are available upon request due to privacy/ethical restrictions. Data that support the findings of this study are available upon request from the corresponding author. The data are not publicly available due to privacy or ethical restrictions.
